# SNP-Based Analysis Reveals Authenticity and Genetic Similarity of Russian Indigenous *V. vinifera* Grape Cultivars

**DOI:** 10.3390/plants10122696

**Published:** 2021-12-08

**Authors:** Dmitriy Y. Fedosov, Aleksey A. Korzhenkov, Kristina O. Petrova, Alexey O. Sapsay, Fedor S. Sharko, Stepan V. Toshchakov, Adelina A. Kolosova, Elizaveta D. Bakhmutova, Maxim V. Patrushev

**Affiliations:** National Research Centre “Kurchatov Institute”, 123182 Moscow, Russia; oscypek@yandex.ru (A.A.K.); Petrova_KO@nrcki.ru (K.O.P.); sapsay-al@mail.ru (A.O.S.); fedosic@gmail.com (F.S.S.); stepan.toshchakov@gmail.com (S.V.T.); adelinaantnenk@rambler.ru (A.A.K.); bahmutovaelizaveta@gmail.com (E.D.B.); maxpatrushev@yandex.ru (M.V.P.)

**Keywords:** *Vitis vinifera*, autochthonous varieties, Russia, genetic identification, genetic diversity, genetic structure, parentage, SNP, phylogenetics, grapevine genetics, cultivars

## Abstract

9 Russian *Vitis vinifera* grape varieties and the European variety Muscat Hamburg were sequenced and genotyped using 527 SNPs (single nucleotide polymorphisms) with high minor allele frequency for the first time. The data were coupled with previously identified genotypes of 783 varieties and subjected to parentage and population analysis. As a result, contrary to the historical and ampelographic data published in many sources from 1800 to 2012, only two of the nine Russian varieties (Pukhlyakovskiy Belyi and Sibirkovyi) were related to foreign ones and were obviously imported from Europe to the Russian Empire. The remaining seven varieties, led by Krasnostop Zolotovskiy, are not directly related either in the Caucasus or in Europe, they form separate clusters on the genetic distance-based dendrogram and the world parentage network of *V. vinifera*. The resulting pedigree of Muscat Hamburg and its descendants is in accordance with SSR-based (simple sequence repeats) studies and the described pedigree of this variety which confirms the use of the reduced SNP set for further studies.

## 1. Introduction

Since the genome of *V. vinifera* (Pinot Noir variety) was first sequenced [1], genotyping and genome-wide sequencing of different varieties has become a new challenge of modern genetics. The genetic differences between varieties are significant [2], and that is why it is impossible to obtain a complete *V. vinifera* germplasm without studying the varieties from various countries and regions of the world. By 2021 major wine-growing countries of Western Europe had carried out genotyping of their grape germplasm, primarily to identify autochthonous varieties. Now it is time to study the genomics of autochthonous varieties in Eastern Europe, which has started in Serbia [3], Croatia [4], Bosnia and Herzegovina [5], Georgia [6] and other countries. In this way, a picture of the diversity of grape varieties is gradually being formed and questions of their origin are being solved: in addition to purely genetic aspects, these studies help answer questions of history and ethnology.

The first method was the genotyping of varieties by a set of SSR markers, the alternative is fingerprinting with a set of single nucleotide polymorphism (SNP) markers [7]. The number of markers varies from methodology to methodology, at the same time, there is a basic study with a database of 783 varieties genotyped with a set of 10 K SNPs [8]. Despite the fact that some of the Russian autochthonous varieties were genotyped in this study, most of them remain unexplored, although winemaking in Russia, as well as interest in its autochthonous varieties, has been awakening in the 21st century.

The Russian wine market has grown dramatically over the last 10 years [9]. In this country which is recognized a successor state of the Soviet Union (the world’s fourth largest producer of wine by volume [10]), winemaking is starting to be reborn after the federal Law on Viticulture and Wine was approved by President Putin in December 2019.

Deloitte’s 2019 CIS report states that around 60% of Russians consume wines, while only 36% prefer vodka [11]. The winemaking industry’s reform launched in 2013 already led to new vineyard classification. There are now nine winemaking zones in southern Russia, controlled by the state as protected designations of origin (Zashchishchyonoye geograficheskoye ukazaniye, ZGU): Don Valley (Rostov region), Lower Volga (Volgograd and Astrakhan regions), Terek Valley (The Kabardino-Balkarian republic), Kuban (Krasnodar region), Stavropolye (Stavropol region), Crimea and Dagestan.

Viticulture in the southern regions of the Russian Federation started centuries before the Greeks colonized Crimea and the Caucasus in the 7th or 6th century BC [12]. Evidence of ancient and medieval viticulture are justified by the archaeologists and historians in the settlements of Scythians, Khazars, Alans, Circassians, castles of Genoese and Byzantines on Black Sea coast. Russian sources of the 17th century mention winemaking practices of Terek Cossacks in the Eastern Caucasus and Orthodox monks in Astrakhan (Lower Volga), and 18th-century viticulture flourished in the Don Valley [13].

Resulting from the centuries of popular selection, indigenous varieties are the property of a particular nation and culture. Russian indigenous or autochthonous varieties have mainly unknown origin, and they appeared as a result of spontaneous popular hybridization in the pre-phylloxera era [4]. The first Russian cultivars were listed by Peter Pallas, a Prussian botanist invited by Catherine the Great in 1767. In his 516-page study of nature, ethnology and agriculture in the southern provinces of Russian Empire [14] Pallas mentioned 39 local varieties of Crimea, 11 in Astrakhan and some varieties of the Don Valley.

Pallas’s follower Peter von Köppen was another Russian scientist of German origin (1793–1864). Von Köppen’s research on winemaking and wine trade in Russia published in 1832 contains descriptions of 176 autochthonous grape varieties: 66 in Crimea, 41 in Astrakhan, 54 in Dagestan and Terek Valley, 15 in Don Valley [15]. He made short descriptions of their phenology, uttering some versions of their origin; some of them are still in common use.

Comte A.-P. Odart de Rilly in “The Universal Ampelography” (1845), a complete description of the then known grape varieties, made his own classification of Russian cultivars [16]. He was in correspondence with Nikolay von Hartwiss, director of Nikita Botanical Garden in Crimea, who described 15 Russian varieties. According to A.-P. Odart, Kokur Belyi, Gimra and other ones were planted in French grape nurseries.

Years later indigenous varieties of Russia were studied in “The Universal ampelography” published in a dozen volumes in Paris in 1901–1910 by P. Viala and V. Vermorel e.g., with a detailed chapter about Kokur Belyi [17] as well as in “Winemaking in Russia (historical and statistical essay)” by M. Ballas edited in St-Petersburg in 1895–1903 [18].

While studying the autochthonous cultivars [19], Soviet science had a very deep breeding program based on the works of I.V. Michurin: hundreds of interspecific hybrids have been crossed. They displaced autochthonous varieties from plantings throughout the country, a process that lasted in recent decades.

Due to the underdevelopment of local grape nurseries, in the 21st century many enterprises actively import cuttings from Europe. Thus the French varieties such as Cabernet Sauvignon, Chardonnay, Sauvignon Blanc became the most popular in plantings as well as among consumers. According to the International Organization of Vine and Wine (OIV), one-third of plantings all over the world are occupied by 13 cultivars and one-seventh by the top-three varieties including Cabernet Sauvignon [20]. The globalization of viticulture is even more obvious in Russia.

The indigenous cultivars are not so common in modern Russia: e.g., Kokur Belyi occupied 918 ha in 2010 (720 ha in 2020 according to our own data, primarily in Crimea), and Krasnostop Zolotovskiy was planted on 512 ha in 2016. Together, the indigenous Russian cultivarsoccupy no more than 2000 hectares, which is less than 2% of 95,000 ha under vines according to the data of the Russian ministry of agriculture [21]. Nevertheless, these varieties give some expensive and reputed wines.

The systematic study of Russian indigenous varieties started in 2020 after the creation of the Kurchatov Genomics Center. For the first time in history, Russian varieties became the subject of state interest within the framework of the Federal Scientific and Technical Program for the Development of Genetic Technologies. The study of Russian autochthonous grape varieties is designed to put an end to the questions of their origin, to start in-depth study of their germplasm, transcriptomics and metabolomics.

## 2. Results and Discussion

### 2.1. Genetic Characterization of Russian Indigenous Cultivars

DNA sequencing and read pre-processing resulted in 79 million paired-end reads or 22.6 billion nucleotides per sample on average (detailed information in Table 1).

Pairwise IBS-distances were calculated for both SNP sets. Several statistical properties were estimated and median values for all of them are higher in the smaller SNP set, which may lead to better resolution of close varieties (Table 2).

On the basis of the IBS-distances it was proposed that the Pukhlyakovskiy Belyi specimen belongs to the Coarna Alba variety (distance = 0.0047). The Sibirkovyi specimen shows close relation to the Sibirkovyi variety. Greater IBS-distance (0.051) may be a result of DNA degradation and consequent sequencing bias. IBS-distance between Tsimlyanskiy Chernyi and Plechistik specimens equals 0.1148. This value is significantly less than the median value in the whole dataset and indicates relatedness of the specimens. Genotypes of all other specimens have greater distance one from another or from the dataset of Laucou et al. The complete dendrogram of 793 varieties, including 10 from this study and 783 acquired from Laucou et al. SNP set is represented on the Figure S1.

The results for the Pukhlyakovskiy Belyi confirm that it was brought to the Don Valley (obviously before 1832, when it was first mentioned [15] (p. 145) and is not, as was stated, an older local variety [13] (p. 331). This analysis proved its complete identity with the variety Coarna Alba from Romania and Moldova. At the same time, the variety Sibirkovyi is closely related to Pukhlyakovskiy Belyi, as was proved by the analysis based on six SSR markers [22].

As for the other Don varieties (Krasnostop Zolotovskiy, Tsimlyanskiy Chernyi, Varyushkin, Plechistik and Kumshatskiy Belyi) our analysis demonstrates their complete identity and the absence of any direct links with the studied Western European, Caucasian, and Balkan varieties. This completely refutes the previous versions of their origin from the varieties imported to Russia.

While von Köppen modestly assumed that the Don Cossacks could have imported their varieties from France [15] (p. 146) during the occupation of 1814 (the Napoleonic Wars), in 1888, a certain S. Popov from the Don region stated that Plechistik was brought from Epernay (Champagne), and Tsimlyanskiy Chernyi from the Rhine Valley in the early 1700s [23].

M. Ballas, in his 1895 paper, directly suggested that Tsimlyanskiy Chernyi is nothing but Oporto noir (Portugieser), and Krasnostop Zolotovskiy is a local name for Oporto Rouge (Portugieser Rot). Ballas was absolutely sure that all of the cultivars of Don Valley were imported from Western Europe and Balkans, having got in Russia another names [18] (pp. 137–138).

As for Kokur Belyi, all the sources [14,15,16,17,18] from 1800 to 2012 repeated the version of its provenance from Corfu (Ionic archipelago), based on the Greek name of the island—Korkira.

Having previously assumed that the name may have other, Crimean Tatar, Hungarian or Abkhazian roots [24], we can now state that Kokur Belyi is at a great distance from Kakotryghis, the main white variety of the island of Corfu, as well as from any other studied by Laucou et al. cultivar of Greece.

### 2.2. Parentage Analysis

According to current data it is impossible to assume the origin of the Sarkel 1 specimen, despite the presence in the data set of genotypes of the varieties’ alleged progenitors: Plechistik and Kokur Belyi. Nevertheless the historical evidence may confirm its provenance from the vineyards planted before 1953.

At the same time, for the Tsimlyanskiy Сhernyi specimen planted in 1983 the best possible pair of parents is Kokur Belyi × Sarkel 1, giving four Mendelian errors for 126 SNPs. The presence of these errors can be either a consequence of the low quality of the data obtained due to the degradation of DNA in the biomaterial, or of a more complex history of the origin of this specimen. In the first case we can propose that the Sarkel 1 specimen belongs to the Plechistik variety.

Previously, on the basis SSR data variety Tsimlyanskiy Chernyi was predicted as a progeny of Plechistik and Kokur Belyi; Plechistik as a progeny of Tsimlyansky Belyi and Krasnostop Zolotovskiy, Starinky as a progeny of Plechistik and Ekim Kara Faux [25]. Our findings support such a pedigree only in the first case, if we assume in all cases may be caused by the presence of different varieties under the same name Plechistik. The Ekim Kara Faux variety was not genotyped in study of Laucou et al., but the parent–offspring relation between Plechistik and Starinky was not identified.

The obtained data conforms the results of SSR-typing indicated in the VIVC database for the following varieties analyzed by us: Muscat Hamburg and Kokur Belyi (Table 3).

Further analysis revealed several possible parent–offspring relations, the relations of Russian varieties are presented in Table 4. Despite possible PO relations between Tsimlyanskiy Chernyi, Kokur Belyi and Plechistik, they did not form any valid trio. To reconstruct their pedigree, more genotyped grape specimens from the Don Valleyare required.

**Figure 1:**
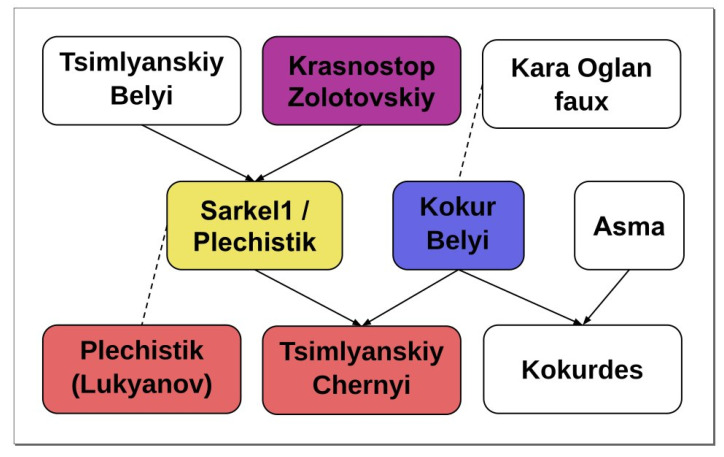
Predicted parent–offspring relations of Russian grape cultivars. White rectangles-data from Lacou et al., 2018. Coloured rectangles-data from the current study. Colors correspond sampling locations from Figure 1. Arrows show directed parent-to-offspring relations, dashed lines show undirected parent–offspring relations.

The reconstructed parentage network based on PO relations includes eight clusters with more than three varieties, six clusters formed by three varieties and 10 PO pairs. The biggest cluster includes 392 varieties-roughly the half of all genotypes included in the analysis, seven clusters including more than three varieties are made up of 38 specimens. Muscat Hamburg, Sibirkovyi and Pukhlyakovskiy Belyi varieties belong to the biggest cluster, Varyushkin variety is a singleton, which does not belong to any cluster, while all other varieties form two clusters. Hypothetical parentage network parts including Russian varieties are shown in Figure 1.

The only foreign variety here is Kara Oglan Faux, a Turkish cultivar from INRA—the French collection. Kara Oglan is another name for Ekşi Kara, old Anatolian variety [26]. However, this variety has a black skin, while the variety published by Laucou et al. in database under the code B00F6O0 is white-skinned. Obviously this was the first reason to add “faux” (false) to cultivar’s name. At the same time in the study published in 2015 by S. Gorislavets et al. from Magarach Institute together with V. Laucou [27], Crimean varieties were genotyped and compared to INRA database using 22 nuclear and 3 chloroplast SSR. Among synonyms found there was ‘Khalil izyum’ = ‘Kara oglan faux’. Khalil izyum is an autochthonous variety of Crimea, it belongs to *V. vinifera* pontica Negr. group [28], which explains the close relationship of the “Kara oglan faux” specimen to Kokur Belyi.

### 2.3. Chlorotypes

Identified chlorotypes are in agreement with data reported previously [29] and available in the VIVC database: B-Varyushkin, C-Kumshatskiy Belyi and Pukhlyakovskiy Beliy, D-Krasnostop Zolotovskiy, Plechictik, Tsimlyanskiy Chernyi, Sarkel 1 and Muscat Hamburg.

### 2.4. Grape Varieties Clustering

**Figure 2:**
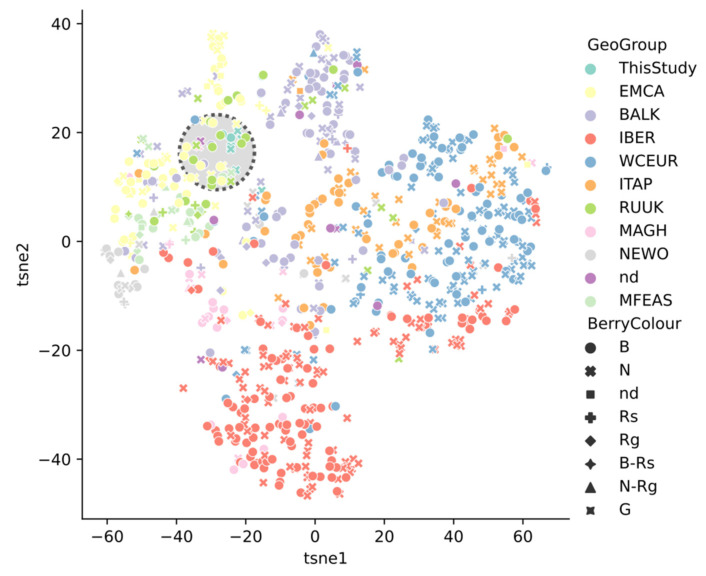
The tSNE visualization of genotype distances. Indigenous Don and Crimean specimens and related varieties are highlighted.

tSNE visualization of genotype distances reveals several clusters (Figure 2) related to the geographic origin of grape varieties and possible routes of their spread.

According to their origin, the SNP-portraits were stratified into nine geographic regions according to previously publishedclustering [8,30]. They include Middle and Far East (MFEAS), Eastern Mediterranean and Caucasus (EMCA), Russia and Ukraine (RUUK), Balkan (BALK), Western and Central Europe (WCEU), Italian Peninsula (ITAP) to Maghreb (MAGH) and Iberia (IBER), and from the New World (NEWO), including America, Australia, New Zealand and South Africa.

As the visualization demonstrates, the varieties considered in this study cluster together with the Don, North Caucasus, and Crimean varieties previously considered by Laucou et al. as RUUK, ranking between the Eastern Mediterranean/Caucasian EMCA and Balkan clusters BALK. They are located at a fairly large distance from both Western European (France, Germany, Austria, etc.) and Iberian, Italian varieties. As noticed, Pukhlyakovskiy Belyi and Sibirkovyi take part of Balkan cluster, to which their homologue/relative Coarna Alba belongs.

### 2.5. ADMIXTURE Analysis

ADMIXTURE analysis gave the best results with four possible ancestral populations (K = 4), referred as AP1-AP4. Only 49 of 793 genotyped grape specimens (6.2%) were assigned to a single ancestral population (AP) using the 80% threshold: AP1 contains 23 specimens, AP2-4, AP3-16, AP4-6 respectively. The results of STRUCTURE analysis on the full 10K SNP dataset [8] showed the same number of most likely APs, and at the same time more specimens (30%) were assigned to a single AP. The difference may be caused by inequality in data processing by ADMIXTURE and STRUCTURE or by usage of the reduced SNP dataset. Anyway, the APs resulted in both analyses representing highly similar groups: wine grape varieties from the West (AP1), table grape varieties from the East (AP3), wine-table grape varieties from the Iberian Peninsula (AP4), and wine grape varieties from the Balkan region (AP2) [8]. AP1 and AP2 demonstrate the division of European grape cultivars into Frankish (or Noble) and Hunnic groups. AP1 includes among others Gros Manseng, Deckrot, Manseng Noir, Pinot Noir, Beclan, Savagnin Blanc, Persan. At the same time Javor Weiss, Furmint, Heroldrebe, Gouais Blanc (Heunisch Weiss) were attributed to AP2. The last one, Gouais Blanc, had been proposed as a possible grape cultivar brought to the territory of modern France by Roman Emperor Marcus Aurelius Probus [31].

**Figure 3:**
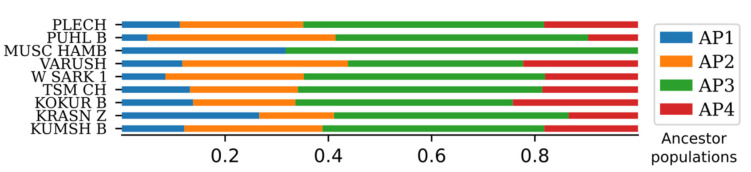
ADMIXTURE analysis (K = 4) for ancestry estimation.

All grape specimens genotyped in our study showed sufficient influence from all four APs excepting Muscat Hamburg (Figure 3). This cultivar most probably inherits 32% of genetic material from AP1 (Western Europe) and 68% from AP3 (Eastern grapevines), which is in good accordance with its origins from Schiava Grossa (northern Italy) and Muscat d’Alexandrie (Egypt). Russian cultivars do not demonstrate any dominant AP. But the AP3 (“Eastern”) is most present: from 34% in Varyushkin to 49% in Pukhlyakovskiy Belyi (which was determined as a synonym for Coarna Alba). For Tsimlyanskiy Chernyi—an indigenous Don grape cultivar—AP3 is responsible for 47% of the genetic material. AP2 (“Hunnish”) represents from 15% (Krasnostop Zolotovskiy) to 32% (Varyushkin) of genetic material in Russian cultivars and 36% for Pukhlyakovskiy Belyi. AP4 (“Iberian”) varies from 9% in Pukhlyakovskiy Belyi and 13% in Krasnostop Zolotovskiy to 24% in Kokur Belyi. The least represented AP1 (“Frankish”) varies from 5% in Pukhlyakovskiy Belyi and 8% in Sarkel 1 to 27% in Krasnostop Zolotovskiy. Previously studied Russian and Soviet grapevine varieties Starinky, Tsimlyanskiy Belyi, Lialmigui (Moldavia), Shabash, Kolossie Gebirgig (Ukraine) have highly similar patterns of distribution of APs. Other cultivars from RUUK cluster may be attracted to Eastern ancestry: Kizil Yakdona, Rannii Vira, Shtur Angur, Ak Ouzioum Tagapskii, Dili Kaftar, Asma etc., some of them represent strong European influence: Gros Colman, Granatovyi, Sorok Let Oktiabria, Bastardo Magaratchskii, Tchainak = Gordin Verde, Mourvedre Goule, etc. This can be explained by their provenance from European cultivars crossed with Soviet ones. The complete ADMIXTURE analysis results based on this study and varieties acquired from Laucou et al. SNP set are represented on the Figure S2.

## 3. Materials and Methods

### 3.1. Plants and Sampling

**Figure 4:**
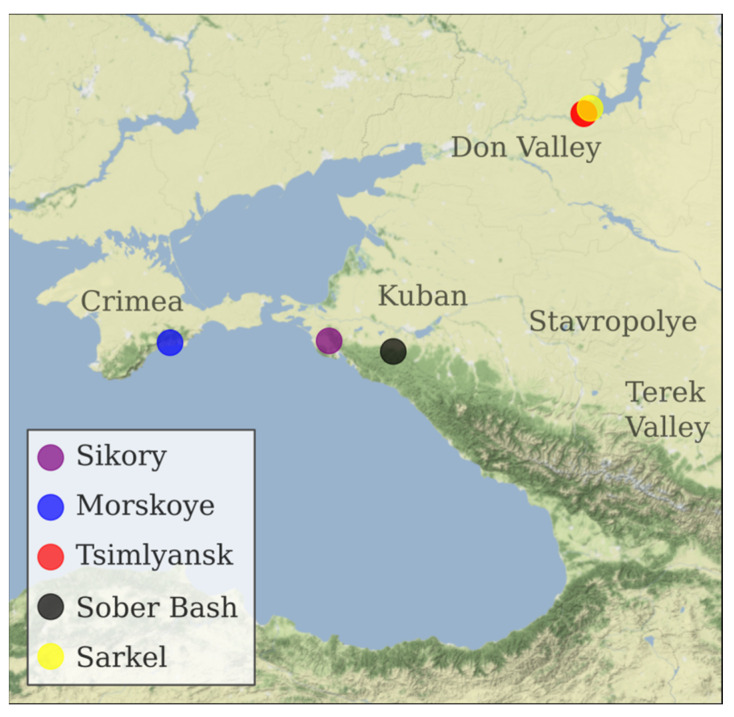
Geographical location of vineyards in southern Russia where the 10 grapevine samples included in the study were recovered.

Plant samples were collected in vineyards of Southern Russia (Table 5 and Figure 4). Sarkel 1 (Wild Grape) sample was collected on the site of the former vineyards of the stanitsa (Cossack village) Tsimlyanskaya. Along with dozens of other settlements, it was flooded to give place to the Tsimlyansk reservoir on Don River in 1953. Since then the eroded slopes are no longer in use for vineyards. Muscat Hamburg from N. Lukyanov’s vineyard in Tsimlyansk was taken as a reference specimen with a known pedigree for the verification in parent–offspring analysis.

### 3.2. DNA Isolation

DNA extraction was performed using the protocol based on a modified cetyl trimethylammonium bromide (CTAB) extraction procedure [32], allowing the rapid DNA extraction from small amounts of leaf material without employment of liquid nitrogen for the initial tissue. Purity of DNA from protein and polysaccharide contamination was confirmed by A260/280 and A260/230 ratios calculated from the spectrophotometric readings using NanoDrop1000 (Thermo Scientific, Waltham, MA, USA). DNA concentrations were measured using Qubit 3.0 fluorometer (Life Technologies, Carlsbad, CA, USA).

### 3.3. DNA Sequencing

Paired-end DNA libraries were prepared according to the NEBNext Ultra II DNA Library Prep Kit for Illumina protocol (New England Biolabs, Ipswich, MA, USA). Their quality and fragment lengths were evaluated using the Agilent Bioanalyzer 2100 (Agilent Technologies, Santa Clara, CA, USA) using the High-Sensitivity DNA kit (Agilent Technologies, Santa Clara, USA).

DNA sequencing was performed on Illumina Novaseq 6000 sequencer (Illumina, San Diego, CA, USA) using S2 flowcell (Illumina, San Diego, USA) and reagent kit (Illumina, San Diego, USA) read length was 150 nucleotides.

### 3.4. NGS Data Processing and Genotyping

Reads were trimmed by quality and adapter sequences were removed with BBduk [33] minimum quality was set to 18, all other parameters were set to default. Reference genome assembly of *V. vinifera* was downloaded from NCBI RefSeq database, accession GCF_000003745.3 [1]. Reads were mapped onto the reference genome with bowtie2 [34], mapping files were processed with samtools [35], variant calling, genotype extraction and consensus sequence creation were performed with bcftools [35]. The cholorotypes of the specimens were determined from the chloroplast consensus sequences [29].

A reduced set of SNPs was acquired from Laucou et al. SNP set [8] (detailed information is represented in Table S1). Only SNPs with minor allele frequency (MAF) > 0.45 were selected. SNP coordinates were verified using a homology search of flanking sequences with BLAST against GCF_000003745.3 genome assembly. SNPs with ambiguous flanking sequences were discarded. SNPs with coordinates differing from those specified were discarded. SNPs located in less than 150 bp from a genome locus marked as a repeat region in the RefSeq annotation were discarded.

To estimate genetic distance between specimens, the sum of pairwise Hamming distances between genotypes was divided by the number of SNP. The resulting value equals 1–IBS (identity-by-state) and can be called IBS-distance,

$$Dist\left(i,j\right)=\frac{\sum_{k}^{N}Dist_{Hamming}\left(G_{k}^{i},G_{k}^{j},\right)}{N},$$

where $G_{k}$ is a genotype at *k*-th of *N* loci.

Pairwise IBS-distances were subjected to hierarchical clustering with SciPy library using unweighted pair group method with arithmetic mean (UPGMA). The distance dataset was also subjected to dimensional scaling using tSNE [36] and subsequent visualization with seaborn library v0.11.2.

### 3.5. Population Analysis

Parentage analysis was made as described earlier [37], the algorithm was reimplemented on Python3, source code is available at https://github.com/laxeye/russian-grape-cultivars-genotyping (accessed on 6 December 2021). Shortly, for all combinations of parent homozygous genotypes expected progeny (EP) were predicted. The Gower dissimilarity metric was used to assess distance between predicted offspring (PO) and EP. The significance of resulting trios was tested with the Dixon test. Identified parent-offspring relations were visualised with Cytoscape [38].

ADMIXTURE analysis v1.23 was performed with a default five-fold cross-validation (--cv = 5) based on 527 SNPs. The number of ancestor populations was estimated from K = 2 to K = 9 in 100 bootstraps with different random seeds. The analyzed set included varieties from Laucou et al. database.

## 4. Conclusions

A limited set of SNPs has proven to be a reliable tool for determining the distances between varieties and parent–offspring relationships. The present study demonstrated that the examined Russian autochthonous grape varieties are divided into two groups: (1) the smallest (two of nine): imported from Europe (Pukhlyakovskiy Belyi and his probable descendant Sibirkovyi); (2) the largest (seven of nine): having no direct proven links with varieties of Europe and the Caucasus. Moreover, these varieties form their own internal cluster with parent–offspring trios and duos.

Kokur Belyi might play a crucial role in the emergence of some autochthonous varieties of southern Russia. Considering the results of genome analysis in this study, it may be appropriate to recall the first results of ampelographic studies of A.-P. Odart and N. Hartwiss, who referred the varieties of the Black Sea region to the Kokur family (Tribu des Kokur) [39].

To complete the genesis of autochthonous varieties of Russia, a more detailed study of both wild grapes and other autochthonous varieties is required. It should include the genetic research of varieties from neighboring Don Valley regions and countries (Dagestan, Abkhazia), as well as wild grapes on the sites of pre-Soviet and late-Soviet plantings that may represent some lost autochthonous varieties and the ancestors of modern ones.

## Figures and Tables

**Table 1:** Grapevine whole genome sequencing results.

ID, Cultivar Name	Read Pairs after Trimming, M	Bases after Trimming, B	Bases Mapped, B	Alignment Rate, %	Mean Coverage
KRASN_Z Krasnostop Zolotovskiy	56.2	15.8	14.4	90.77	29.58
KOKUR_B Kokur Belyi	66.4	18.8	17.1	91.20	35.25
TSM_CH Tsimlyanskiy Chernyi	75.3	21.7	5.7	26.33	11.77
W_SARK_1 Sarkel 1 (Wild grape)	120.4	34.4	12.0	34.72	24.6
VARYUSH Varyushkin	51.9	14.6	13.1	89.97	26.97
SIBIRK Sibirkovyi	80.8	23.2	4.7	20.22	9.65
KUMSH_B Kumshatskiy Belyi	95.9	26.9	23.3	86.42	47.86
PLECH Plechistik (Lukyanov)	80.1	11.0	9.7	88.45	19.94
PUHL_B Pukhlyakovskiy Belyi	133,3	19.2	18.3	95.01	37.57
MUSC_HMB Muscat Hamburg	129.3	18.6	17.5	94.43	36.06

Note: In our article we use the common rules of transliteration of Russian names into English. For example https://usefulenglish.ru/vocabulary/russian-names-in-english-en (accessed on 2 December 2021). At the same time, other cited sources (Laucou et al., Lacombe et al., VIVC database) sometimes use other transliterations of Russian varieties, including German one (Tzimliansky).

**Table 2:** Median values of statistical properties of genotype distance distribution for two single nucleotide polymorphism (SNP) sets.

IBS-Distance.	SNP Set Size	
	10 K	527
median	0.2572	0.3705
mean	0.2573	0.3687
min	0.1568	0.2372
max	0.3171	0.4573
stdev	0.0195	0.0272

**Table 3:** Supposed novel and confirmed trios of *V. vinifera* varieties.

Offspring	Parent 1	Parent 2	Offspring-ExpProgeny Gower Distance, %	Mendellian Errors/Loci	Previously Reported
Muscat Hamburg	Schiava Grossa ~ Trollinger~Frankenthal *	Muscat d’Alexandrie	0	0/133	Lacombe et al., 2013
Alphonse Lavallée	Muscat Hamburg	Gros Colman = Dodrelyabi	0	0/112	Lacombe et al., 2013
Roumi Noir	Muscat Hamburg	Darkaia Noir = Coarna Neagra	0.85	1/118	Lacombe et al., 2013
Misket Rusenski	Muscat Hamburg	Cardinal	0	0/144	Lacombe et al., 2013
Italia	Muscat Hamburg	Bicane	0	0/125	Lacombe et al., 2013
Kokurdes Belyi	Kokur Belyi	Asma	0.77	1/130	Lacombe et al., 2013
Tsimlyanskiy Chernyi	Kokur Belyi	Sarkel-1/Wild grape **	3.17	4/126	Lacombe et al., 2013

Note: ~ stands for incorrect synonym, = stands for valid synonym. * B00EQX0 = Frankenthal (incorrect synonym of Schiava Grossa used by Laucou et al.). ** Sarkel 1 may be considered as Plechistik.

**Table 4:** Undirected parent–offspring relations.

Variety 1	Variety 2	Mendelian Errors/Loci	Previously Reported
Tsimlyanskiy Chernyi	Kumshatskiy Belyi	5/155	-
Sarkel 1/Wild Grape	Krasnostop Zolotovskiy	0/141	Lacombe et al., 2013
Sarkel 1/Wild Grape	B00EQSE (Starinky)	0/135	Lacombe et al., 2013
Sarkel 1/Wild Grape	B00EQSJ (Tzimliansky Belyi)	1/137	Lacombe et al., 2013
Kokur Belyi	B00F6O0 (Kara oglan faux)	3/153	Lacombe et al., 2013

**Table 5:** Genotyped *V. vinifera* specimens.

Specimen ID	Variety	Berry Color	Origin
KRASN_Z	Krasnostop Zolotovskiy	Black	Sikory Estate, Novorossiysk, Krasnodar region (ZGU Kuban.Novorossiysk). Planted in 2014.
KOKUR_B	Kokur Belyi	White	Morskoye vineyard of Massandra winery, Sudak district, Republic of Crimea (ZGU Crimea). Planted in 1978.
TSM_CH	Tsimlyanskiy Chernyi	Black	Pavel Serikov vineyard, Tsimlyansk, Rostov region (ZGU Don Valley). Planted in 1983.
W_SARK_1	Sarkel 1/Wild Grape	Black	Sarkel village, Tsimlyansk district, Rostov region.
VARYUSH	Varyushkin	Black	Sober Bash Vinery, Severskiy district, Krasnodar region (ZGU Kuban. Afips valley). Cuttings from A. Zarechenskiy nursery, Rostov region. Planted in 2013.
SIBIRK	Sibirkovyi	White	Pavel Serikov vineyard, Tsimlyansk, Rostov region (ZGU Don Valley). Planted in 1983.
KUMSH_B	Kumshatskiy Belyi	White	Pavel Serikov vineyard, Tsimlyansk, Rostov region (ZGU Don Valley). Planted in 1983.
PLECH	Plechistik	Black	Nikolay Lukyanov vineyard, Tsimlyansk, Rostov region (ZGU Don Valley). Planted in 1983.
PUHL_B	Pukhlyakovskiy Belyi	white	Nikolay Lukyanov vineyard, Tsimlyansk, Rostov region (ZGU Don Valley). Planted in 1983.
MUSC_HMB	Muscat Hamburg	black	Nikolay Lukyanov vineyard, Tsimlyansk, Rostov region (ZGU Don Valley). Planted in 1983.

## Data Availability

The original data presented in this research work are stored in the databases of National Research Centre “Kurchatov Institute”.

## Supplementary Materials

The following are available online at https://www.mdpi.com/article/10.3390/plants10122696/s1, Table S1: Set of 527 SNPs used in this study, Figure S1: complete dendrogram of 793 varieties, including 10 from this study and 783 acquired from Laucou et al. SNP set, Figure S2: complete ADMIXTURE analysis results based on this study and varieties acquired from Laucou et al. SNP set.
