# Supplementary material for: SNP-Based Analysis Reveals Authenticity and Genetic Similarity of Russian Indigenous V. vinifera Grape Cultivars
Source: Plants (Basel). 2021 Dec 8;10(12):2696. doi: 10.3390/plants10122696 (PMC8706836; doi:10.3390/plants10122696)

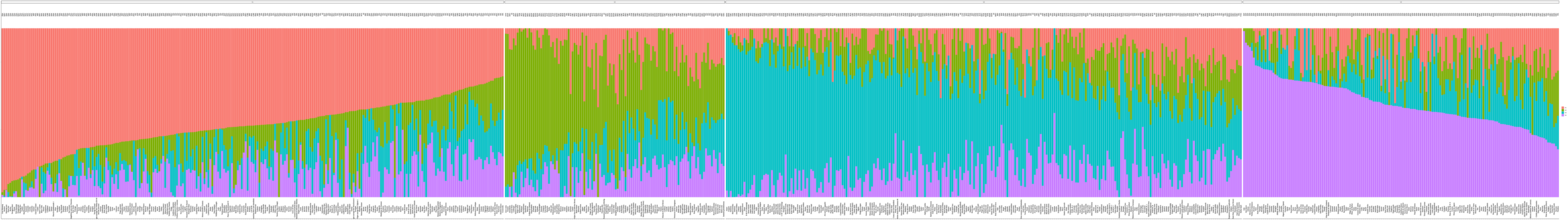

Supplement: Supplementary file 1 [file Figure S 2.pdf › Figure S 2.pdf]
